# Epidemiology, frost resistance, and emerging species-specific diagnostics for Trichinella nativa: Current evidence and future priorities

**DOI:** 10.14202/vetworld.2026.3793-3822

**Published:** 2026-08-27

**Authors:** Aibek Zhumalin, Aisarat Gajimuradova, Saulet Issayev, Alfiya Syzdykova, Fariza Zhagipar, Nurtai Gubaidullin, Nasipkhan Askarova, Catalina Picasso-Risso, Ainur Ibzhanova, Zhannara Akanova, Assem Jangulova, Aizada Mukhanbetkaliyeva, Orken Akibekov

**Affiliations:** 1 Institute of Animal Science and Veterinary, Saken Seifullin Kazakh Agrotechnical University, 62 Zhenis Avenue, Astana 010000, Kazakhstan.; 2 Engineering Center for Organic Agrotechnology, Seifullin University Agrotechnopark, Saken Seifullin Kazakh Agrotechnical University, 62 Zhenis Avenue, Astana 010000, Kazakhstan.; 3 Department of Large Animal Clinical Sciences, College of Veterinary Medicine, Michigan State University, East Lansing, MI 48824, USA.; 4 Institute of Engineering and Food Technology, Saken Seifullin Kazakh Agrotechnical University, 62 Zhenis Avenue, Astana 010000, Kazakhstan.; 5 Department of Biosystems and Agricultural Engineering, Michigan State University, East Lansing, MI 48824, USA.; 6 Joint Kazakh–Chinese Laboratory for Biological Safety, Saken Seifullin Kazakh Agrotechnical University, 62 Zhenis Avenue, Astana 010000, Kazakhstan.; 7 Department of Technology and Standardization, Faculty of Technology, K. Kulazhanov Kazakh University of Technology and Business, 37A K. Mukhamedkhanov Street, Astana 010008, Kazakhstan.

**Keywords:** Arctic wildlife, Central Asia, epidemiology, molecular diagnosis, recombinant antigens, Trichinella nativa, trichinellosis, wildlife surveillance

## INTRODUCTION

Trichinellosis (trichinosis) is a globally distributed zoonotic helminthic disease that has been recognized for more than two centuries, although paleopathological evidence suggests that human infection dates back at least 3,500 years [1]. According to international surveillance reports, 65,818 cases of trichinellosis and 42 associated deaths were officially reported in 41 countries between 1986 and 2009, highlighting the long-standing public health importance of this disease [2]. Although these data represent only officially documented cases over a 23-year period, recent estimates from the Centers for Disease Control and Prevention indicate that approximately 10,000 cases of trichinellosis occur worldwide each year, suggesting that the true disease burden remains substantially underestimated despite declining incidence in several countries [3]. *Trichinella* spp. continue to circulate on every continent except Antarctica, with numerous natural and synanthropic transmission foci maintaining the global persistence of the disease [4].

Recent epidemiological data further emphasize the continuing public health relevance of trichinellosis. Joint reports from the European Food Safety Authority and the European Center for Disease Prevention and Control documented 96 confirmed cases in European Union member states during 2019, with 79.2% of cases occurring in Bulgaria, Italy, and Spain, corresponding to an incidence of 0.02 cases per 100,000 population [5]. Subsequently, 76 confirmed cases were reported in 2023, rising to 102 in 2024 and 114 in 2025, indicating a gradual upward trend in multiregional surveillance alerts and reinforcing the need for improved epidemiological surveillance and early diagnostic strategies [6, 7]. Trichinellosis also remains an important zoonosis in Asia. Between 2004 and 2009, China reported 15 outbreaks involving 1,387 human cases and four deaths, primarily associated with pork consumption [8]. Similarly, more than 1,600 human cases were reported in Thailand, Vietnam, and Laos between 2001 and 2021, predominantly linked to the traditional consumption of raw or undercooked meat [9]. These regional differences reflect variations in dietary habits, wildlife exposure, food preparation practices, and veterinary control programs. Moreover, climate change is increasingly influencing the spatial distribution of *Trichinella* spp. Rising temperatures in Arctic and subarctic regions may facilitate northward expansion of wildlife reservoirs, alter sylvatic transmission dynamics, and contribute to the emergence of infection in previously non-endemic areas.

A 10-year surveillance study conducted in Kazakhstan (2012–2021) identified trichinellosis in 247 of 1,372 wild carnivores (18.0%). The highest prevalence was observed in badgers (66.7%; 6/9), red foxes (22.0%; 50/227), wolves (20.5%; 83/405), and steppe foxes (18.2%; 11/60). Infected animals were detected across 10 administrative regions, demonstrating widespread circulation of the parasite. Molecular characterization confirmed the predominance of *Trichinella nativa*, indicating that this species is the principal etiological agent circulating within Kazakhstan's natural transmission foci [10]. These findings are consistent with observations from other Arctic and subarctic ecosystems, highlighting the remarkable ecological adaptability and stable transmission cycles of *T. nativa* [4].

The global distribution and epidemiological diversity of trichinellosis largely reflect the considerable biological and genetic diversity of the genus *Trichinella*. Currently, the genus comprises 10 recognized species and 15 genotypes, which are broadly classified into encapsulated species (*T. spiralis*, *T. nativa*, *T. britovi*, *T. murrelli*, *T. nelsoni*, *T. patagoniensis*, *T. chanchalensis*, and the recently recognized genotypes T14 and T15) and non-encapsulated species (*T. pseudospiralis*, *T. papuae*, and *T. zimbabwensis*) [11]. Nevertheless, the taxonomy of the genus continues to evolve because genotypes T6, T8, and T9 remain under consideration as potential distinct species, illustrating the complex phylogenetic relationships within the genus [4].

Although the taxonomic diversity of *Trichinella* has important ecological and evolutionary implications, the clinical course of human trichinellosis is generally similar regardless of the infecting species. Infection occurs following ingestion of meat containing viable encapsulated larvae. After release in the stomach, larvae invade the small intestinal epithelium, undergo four successive molts, and mature into adult worms. During the intestinal phase, which typically lasts about 1 week, patients often develop gastroenteritis. Subsequently, female worms release up to 1,500 larvae that migrate through the circulation and encyst within striated skeletal muscles over the following 2–3 weeks, resulting in fever, myalgia, edema, and eosinophilia [12].

Despite the relatively uniform clinical manifestations of human trichinellosis, the epidemiology of the disease varies considerably with the ecology, taxonomy, and transmission dynamics of the infecting species. These ecological differences determine the principal reservoir hosts, transmission routes, and regional outbreak patterns, emphasizing the importance of understanding the geographic distribution and natural circulation of *Trichinella* spp. [13–15].

Domestic pigs remain the principal source of human trichinellosis worldwide because *T. spiralis* is highly adapted to the pig-human transmission cycle and has historically accounted for most human infections. However, improvements in veterinary inspection and commercial pig production have progressively shifted the epidemiological importance toward sylvatic transmission in many regions. In Europe, outbreaks are increasingly associated with the consumption of wild boar meat, with major outbreaks reported in Bulgaria, Spain, Serbia, and Italy, underscoring the importance of wild boar as a significant wildlife reservoir [4, 13]. In Asia, transmission patterns are more strongly influenced by regional dietary practices. Human infections associated with consumption of wild boar and badger meat have been documented in South Korea, where most outbreaks were linked to the consumption of raw or undercooked wild boar meat [16, 17]. An additional transmission pathway occurs in Arctic marine ecosystems, where *Trichinella* circulates among polar bears, walruses, ringed seals, beluga whales, and killer whales. This marine cycle represents a persistent zoonotic threat for indigenous communities that traditionally consume raw or inadequately cooked marine mammal meat [18–20].

Among the recognized species, *T. nativa* deserves particular attention because of its distinctive biological characteristics and growing public health importance. Unlike other members of the genus, *T. nativa* exhibits exceptional freeze resistance, allowing larvae to remain viable for prolonged periods in frozen wildlife carcasses. Consequently, conventional household freezing cannot reliably inactivate infective larvae, increasing the risk of human infection through consumption of contaminated game meat. In addition to its public health implications, *T. nativa* causes substantial economic losses because infected wildlife carcasses must be discarded following veterinary inspection. Increasing reports describing the expansion of *T. nativa* into new areas of North America and Europe, together with advances in molecular and next-generation sequencing (NGS) technologies, suggest that the current distribution and epidemiology of this species may be underestimated because of mixed infections and limitations of conventional identification methods [21–23].

Integrating regional surveillance data with global observations further demonstrates that Kazakhstan represents the southern limit of *T. nativa* circulation within the Palearctic region, whereas the Nearctic distribution extends southward to approximately 45°–48°N across Alaska and the northern United States. These observations highlight the need to reconsider existing diagnostic paradigms because species diversity within the genus directly influences surveillance accuracy, outbreak investigation, and disease prevention.

Improving diagnostic methods therefore remains a major research priority. Artificial digestion remains the diagnostic gold standard for veterinary meat inspection; however, its sensitivity decreases substantially in low intensity infections and its application is limited in clinical settings, as demonstrated during the 2022 outbreak in the United States [21]. Serological assays based on excretory-secretory (ES) antigens are also constrained by a prolonged serological window and reduced sensitivity during the early intestinal stage of infection [24]. In contrast, the *T. nativa* serine protease recombinant antigen rTnsp-4E has demonstrated earlier detection of infection between 10 and 17 days post-infection and superior diagnostic sensitivity compared with conventional ES antigen-based assays under experimental conditions [25].

Considerable progress has been achieved in developing recombinant diagnostic antigens for *T. spiralis*. Numerous candidates, including rTsSP, rTsTryp, Ts87, Ts14-3-3, and TsPmy, have demonstrated excellent diagnostic performance. Early serological detection has been achieved as early as 7 days post-infection using rTsSP, with reported sensitivity and specificity of 98.1% and 99.5%, respectively [26]. Similarly, rTsTryp detects infection from 8 days post-infection with sensitivity and specificity of 98.1% and 98.7%, respectively [27], whereas Ts87 [28], Ts14-3-3 [29], and TsPmy [30] have demonstrated high diagnostic performance in experimental animal models.

In contrast, the development of species-specific recombinant antigens for *T. nativa* remains extremely limited. Existing heterologous antigens, such as rTbES21 derived from *T. britovi*, produce delayed seroconversion (41–45 days post-infection), substantially limiting their usefulness for early diagnosis [31]. Although rTnsp-4E is one of the most promising candidate antigens, its evaluation has been limited to experimental studies with relatively small sample sizes, lacking multicenter validation or comprehensive assessment of diagnostic sensitivity, specificity, and cross-reactivity [25]. Furthermore, no published studies have evaluated the performance of rTnsp-4E in mixed infections involving *T. nativa*, *T. britovi*, and *T. spiralis*, despite field evidence indicating that mixed infections may account for 10%–25% of natural transmission foci in endemic regions such as Kazakhstan [10]. Collectively, these limitations create a substantial diagnostic gap between *T. spiralis* and *T. nativa* and emphasize the urgent need to develop and validate species-specific recombinant antigens for reliable early diagnosis [23].

Despite the availability of highly sensitive recombinant antigens for *T. spiralis*, validated diagnostic markers for early, species-specific detection of *T. nativa* remain unavailable. The lack of standardized multicenter validation studies, insufficient information regarding diagnostic performance, and limited understanding of cross-reactivity continue to impede the development of reliable serological assays. Consequently, the use of nonspecific antigenic platforms may delay seroconversion by 4–5 weeks compared with assays optimized for *T. spiralis*, creating a critical diagnostic blind period during the earliest stages of infection.

Although considerable advances have been made in understanding the epidemiology and diagnosis of *Trichinella* spp., current knowledge of *T. nativa* remains fragmented and disproportionately limited compared with that of *T. spiralis*. Existing reviews have largely emphasized general aspects of trichinellosis without critically integrating the unique epidemiology, freeze resistance, molecular biology, recombinant antigen development, and diagnostic challenges specific to *T. nativa*. Moreover, no comprehensive review has synthesized recent evidence from Central Asian surveillance programs together with advances in recombinant antigen research, molecular diagnostics, and NGS to identify the principal barriers to early and species-specific diagnosis. This lack of integration has hindered the establishment of standardized diagnostic strategies and limited the translation of emerging molecular discoveries into practical surveillance and clinical applications.

**Figure 1:**
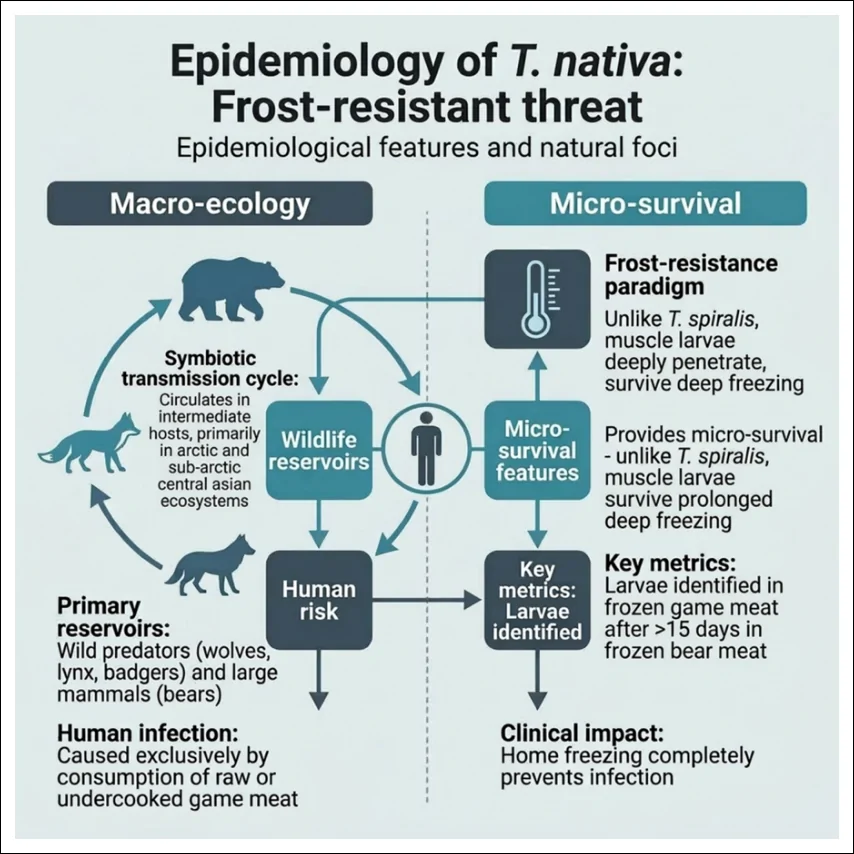
Epidemiology of *Trichinella nativa* showing the natural transmission cycle, major wildlife reservoirs, geographic distribution in Arctic and subarctic ecosystems, and the contribution of freeze-resistant larvae to environmental persistence and foodborne transmission to humans [Source: The Figure was prepared by Orken Akibekov with the assistance of Gemini (Google) for AI-assisted figure generation and Canva for figure design and final layout].

The aim of this review was to critically synthesize and integrate the current evidence regarding the epidemiology, taxonomy, transmission ecology, molecular biology, and diagnostic approaches for *T. nativa*, with particular emphasis on recombinant antigen development and species-specific serological diagnosis. Furthermore, this review evaluates the strengths and limitations of existing diagnostic methods, identifies major knowledge gaps in early detection and molecular surveillance, and highlights future research priorities for the development and validation of reliable species-specific diagnostic platforms capable of improving wildlife surveillance, food safety, and public health in endemic regions. An overview of the topics covered in this review is illustrated in Figure 1. 

## REVIEW METHODOLOGY

### Data sources and literature search strategy

The literature search was conducted systematically on January 23, 2026, using PubMed, Scopus, Web of Science, and Google Scholar. To improve coverage and reproducibility, the websites of relevant journals, including Veterinary Parasitology*, *Parasitology Research*, *and Food and Waterborne Parasitology*,* were manually searched. Official repositories of international organizations publishing methodological and regulatory guidance on trichinellosis, particularly the International Commission on Trichinellosis and the World Organization for Animal Health, were also examined.

In addition, relevant articles published in local journals that were not indexed in the selected international databases were reviewed to capture evidence concerning *Trichinella* spp. in Kazakhstan and neighboring Central Asian countries. Boolean operators were used to combine disease-, host-, geography-, and diagnostic-related search terms. The complete English-language search strategy was as follows:

(“*Trichinella nativa*” OR “*T. nativa*”) AND (“trichinellosis” OR “trichinosis”) AND (“Arctic” OR “wildlife” OR “game meat” OR “Kazakhstan” OR “Central Asia”) AND (“diagnosis” OR “serology” OR “ELISA” OR “recombinant antigens” OR “PCR” OR “multiplex PCR” OR “sequencing” OR “next-generation sequencing” OR “NGS”).

### Study selection and eligibility criteria

The study selection process involved sequential identification, deduplication, title and abstract screening, full-text assessment, and final inclusion. The initial electronic and manual searches yielded 189 records. Studies were evaluated using predefined eligibility criteria.

The inclusion criteria comprised original peer-reviewed research articles, retrospective studies, and official methodological or regulatory guidelines; publications written in English or Russian; and studies presenting data specifically related to *T. nativa* or findings directly relevant to its epidemiology, biology, or diagnosis. No strict publication-date restriction was imposed to ensure the inclusion of foundational taxonomic and biological evidence. Nevertheless, priority was given to studies published between 2010 and 2025 to reflect recent developments in molecular epidemiology and diagnostic technologies.

Duplicate publications, studies in which the findings could not be clearly attributed to *T. nativa*, and reports lacking essential methodological information were excluded. Studies were also excluded when they did not adequately describe the sample size, diagnostic procedures, experimental conditions, reference controls, or criteria used for species identification.

### Methodological quality and risk-of-bias assessment

A modified quality-assessment framework was applied to address the methodological heterogeneity associated with combining wildlife surveillance studies, experimental infection investigations, and laboratory antigen-validation studies. The included studies were evaluated for internal and external validity.

Internal validity was assessed according to the use of molecular confirmation for morphology- or digestion-based identification, the appropriateness of diagnostic reference methods, the inclusion of clearly defined positive and negative controls, and the adequacy of experimental and analytical procedures. External validity was evaluated according to sample size adequacy, host species representation, geographic origin, sampling strategy, and the generalizability of the reported findings.

Epizootiological surveys based exclusively on larval recovery, without molecular species confirmation, were considered to be at high risk of species-misclassification bias. These studies were retained only when relevant to the broader epidemiology of trichinellosis, but were excluded from analyses or maps that specifically attribute infection to *T. nativa*.

### Data extraction and synthesis

Relevant information was extracted on study location, host species, sample size, infection prevalence and intensity, diagnostic method, molecular target, antigen type, analytical or diagnostic performance, and principal epidemiological findings. Because of substantial heterogeneity in study designs, host populations, diagnostic methods, and reported outcomes, the evidence was synthesized narratively rather than quantitatively.

**The extracted evidence was organized into the following thematic domains:** taxonomy and genetic diversity, geographic distribution and epidemiology, pathogenesis and clinical characteristics, serological diagnosis, molecular identification, and promising diagnostic targets. This framework enabled comparison across epidemiological and experimental studies and facilitated the identification of major evidence gaps and priorities for future research.

**THE PLACE OF** *T. NATIVA* **IN MODERN TAXONOMY AND THE GENETIC STRUCTURE OF THE GENUS** *TRICHINELLA*

The genus *Trichinella* comprises a phylogenetically and ecologically heterogeneous group of parasitic nematodes (Nematoda: Trichinellidae) adapted to circulate among a broad range of vertebrate hosts, including mammals, birds, and reptiles. Members of this genus can maintain natural transmission cycles across diverse ecosystems, ranging from Arctic terrestrial environments to coastal marine food webs [32]. The contemporary taxonomic framework of *Trichinella* has been established through the integration of morphological, biological, ecological, and molecular genetic criteria. These criteria include host range, reproductive biology, life cycle characteristics, and variability in nuclear and mitochondrial markers used to differentiate species and genotypes [32–34].

This integrated approach, summarized by Zarlenga *et al.* [35], enabled the recognition of 10 valid species and several genotypes of uncertain taxonomic status by 2020. This taxonomic diversity reflects multistage evolutionary divergence and the development of specialized transmission cycles across distinct biogeographic regions.

**Figure 2:**
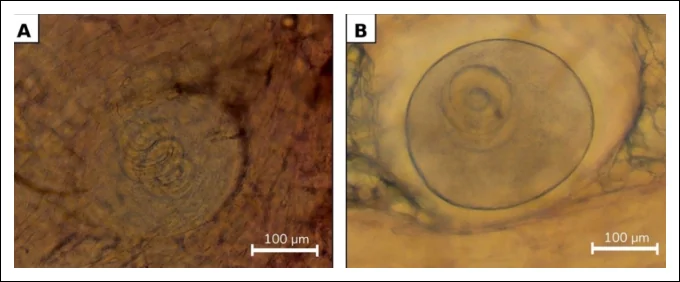
Encapsulated *Trichinella nativa* larvae in the skeletal muscle tissues of naturally infected wild carnivores from the Akmola region, Kazakhstan. (A) Encapsulated *T. nativa* larva in the skeletal muscle tissue of a red fox (*Vulpes vulpes*). (B) Encapsulated *T. nativa* larva in the skeletal muscle tissue of a wolf (*Canis lupus*). The larvae are enclosed within collagenous capsules in striated skeletal muscle, a characteristic feature of encapsulated *Trichinella* species [Source: The figure was prepared by the authors from archival data].

A principal intrageneric taxonomic characteristic of *Trichinella* is the presence or absence of a collagenous capsule surrounding the muscle-stage larva. On this basis, the genus is divided into encapsulated and non-encapsulated clades [36]. Encapsulated species form a distinct collagenous capsule, commonly referred to as the nurse cell–larva complex, which is readily recognized during histological and parasitological examinations (Figure 2).

This distinction has direct diagnostic implications because the sensitivity of routine veterinary meat inspection may depend on the taxonomic identity of the parasite. Non-encapsulated species may be more readily overlooked when detection relies exclusively on conventional morphology-based procedures [37]. High taxonomic resolution is also required for the encapsulated genotypes T6, T8, and T9, which remain classified as genotypes rather than universally accepted species [37]. Genotype T6 is closely related to *T. nativa* and shows evidence of natural hybridization, restricted gene flow, and ongoing evolutionary divergence [38, 39]. Genotype T8 is associated with the *T. britovi* complex and has been identified in wild carnivores in southern Africa, including mixed infections with *T. nelsoni* in lions and leopards [40, 41]. Genotype T9 is primarily associated with Japan, and molecular sequencing confirmed it as the etiological agent of a human outbreak linked to the consumption of undercooked bear meat [42]. Molecular genotyping is therefore necessary not only to distinguish encapsulated from non-encapsulated taxa but also to identify epidemiologically relevant genotypes that cannot be reliably differentiated using morphology alone [43].

The encapsulated clade includes *T. spiralis*, *T. nativa*, *T. britovi*, *T. murrelli*, *T. nelsoni*, *T. patagoniensis*, and *T. chanchalensis*. These species are characterized by the formation of a collagenous capsule around the muscle-stage larva, prolonged persistence in host muscle tissues, and, in some taxa, enhanced resistance to adverse environmental conditions, including low temperatures. These characteristics promote the prolonged preservation of infective larvae within terrestrial and marine food chains [44]. Among these species, *T. spiralis* remains the most extensively investigated because of its worldwide distribution and dominant role in the synanthropic transmission cycle involving domestic pigs. Its historical importance in pig-associated transmission has also led to its widespread use as a reference organism in the development and initial validation of diagnostic assays [45].

*T. nativa* occupies a distinct position among the encapsulated species because its muscle-stage larvae exhibit pronounced cold tolerance and can remain viable for prolonged periods in frozen host tissues. This biological characteristic is particularly important for maintaining trophic transmission in northern ecosystems [46]. Experimental and field observations have demonstrated that infective larvae can persist in frozen game meat for several months. In one study, viable larvae were recovered after 110 days of frozen storage from tissues with high infection intensity exceeding 800 larvae/g, and the parasite was molecularly confirmed to be *T. nativa* [19].

The zoonotic importance of *T. nativa* is supported by documented outbreaks associated with the consumption of wild game in northern North America [21]. During a multistate outbreak investigation conducted in Arizona, Minnesota, and South Dakota in 2022, eight individuals participated in a shared meal that included black bear meat that had been frozen for 45 days and subsequently cooked rare. Six cases of trichinellosis were identified. Notably, two affected individuals had consumed only vegetables cooked with the meat, indicating that cross-contamination contributed to transmission. Motile larvae were recovered from the remaining meat after more than 15 weeks of frozen storage, and molecular testing confirmed *T. nativa* as the causative species [21].

Other encapsulated species, particularly *T. britovi* and *T. murrelli*, are predominantly maintained in sylvatic cycles involving wild mammals. Their zoonotic importance is therefore closely associated with the consumption of infected game meat, resulting in substantial regional variation in the principal sources of human infection [47]. In the northern French Alps, three confirmed and three suspected cases of trichinellosis were reported among six individuals aged 3–69 years after they consumed air-dried wild boar ham in February 2022 [48]. In October 2008, an outbreak in Northern California affected 30 of 38 individuals who attended an event where black bear meat was served. Morphological and molecular examinations identified *T. murrelli* as the causative agent [49].

Non-encapsulated species, including *T. pseudospiralis*, *T. papuae*, and *T. zimbabwensis*, lack a collagenous capsule and exhibit biological characteristics that may influence their detectability during routine examination. Both a sporadic human infection and the first documented outbreak of *T. pseudospiralis* trichinellosis in Thailand during 1994–1995 were associated with the consumption of wild boar meat [50, 51]. Human outbreaks caused by *T. papuae* have also been confirmed in Thailand and Cambodia, demonstrating the substantial zoonotic potential of this species [11, 52]. Human infection with *T. zimbabwensis* has not yet been confirmed; however, the species has been detected in wildlife, including a naturally infected lion, confirming its circulation within African ecosystems [53].

Genotypes T6, T8, and T9 present additional methodological challenges for epidemiological surveillance because they are phylogenetically related to the *T. nativa*–*T. britovi* complex and cannot be distinguished reliably using conventional morphological methods [54]. The taxonomic landscape has also expanded through the description of genotypes T12–T15, including the subsequently recognized species *T. patagoniensis* (formerly T12) and *T. chanchalensis* (formerly T13). Current biogeographic and evolutionary evidence suggests that *T. patagoniensis* may represent an indigenous South American taxon responsible for ancient infections, challenging previous assumptions that the genus was introduced into South America more recently [55].

Among the unnamed genotypes of *Trichinella*, T6, T8, and T9 are of particular epizootiological importance because each has a distinct geographic distribution. Genotype T6 belongs to the North American sylvatic assemblage and has been detected in wild carnivorous and omnivorous mammals in Canada and the United States, including bears, wolves, wolverines, lynxes, and foxes. Recent reviews describe T6 as a cold-adapted, non-Arctic genotype circulating exclusively among North American wildlife [4, 56]. Genotype T8 has a more restricted distribution and has been reported only in South Africa and Namibia, particularly among large wild carnivores such as lions, leopards, and spotted hyenas. Mixed infections involving *T. nelsoni* and T8 have also been documented in lions from Kruger National Park, South Africa [40, 56]. Genotype T9 is considered endemic to Japan and has been identified in Japanese black bears, brown bears, raccoon dogs, red foxes, and raccoons, particularly in Hokkaido and northern Honshu. In addition, T9 has been confirmed as the causative agent of a human trichinellosis outbreak associated with bear meat consumption in Japan [42].

Table 1[19, 21, 23, 35, 45, 47, 54, 57–68] summarizes the principal *Trichinella* species and genotypes, their taxonomic characteristics, geographic distribution, major host groups, and reported occurrence in wild and domestic animals.

**Table 1:** Taxonomic diversity, geographic distribution, principal hosts, diagnostic approaches, and reported infection levels of Trichinella species and genotypes.

**Species/genotype**	**Capsule status**	**Geographic distribution**	**Principal hosts**	**Diagnostic notes**	**Reported prevalence and infection intensity**	**References**
*Trichinella spiralis*	Encapsulated	Cosmopolitan; predominantly associated with synanthropic transmission cycles	Domestic pigs; humans as accidental hosts	ES antigen-based serology; species-specific PCR; ITS-1 amplicon NGS	Farmed wild boars in Jilin Province, China: prevalence = 0.53%; mean intensity = 0.076 ± 0.025 LPG; maximum intensity = 0.21 LPG	[45, 23, 57]
*Trichinella * *nativa*	Encapsulated	Arctic and boreal regions of Eurasia and North America	Wild carnivores; bears are important sources of human infection	Multiplex PCR; PCR-RFLP; ITS-1 amplicon NGS for detecting mixed infections	Wolverines in Yukon, Canada: overall *Trichinella* prevalence = 78%; *T. nativa* = 8%; mixed T6 and *T. nativa* infections = 12%; mean intensity = 22.6 ± 39 LPG; range = 0.1–295 LPG	[19, 21, 54, 58]
*Trichinella * *britovi*	Encapsulated	Europe and Western Asia	Wild boars, badgers, and wild carnivores	PCR targeting ITS regions; NGS-based identification	Martens in Poland: overall prevalence = 17.54%; pine martens = 41.67%; *Martes* spp. = 13.88%; intensity range = 0.17–37.29 LPG; mean intensity = 5.43 LPG	[47, 23, 59]
*Trichinella * *murrelli*	Encapsulated	North America	Wild carnivorous mammals	Molecular identification; ITS-1 amplicon NGS	Bobcats in Oklahoma, USA: prevalence = 5.9% (18/301); 17/18 isolates identified as *T. murrelli*; mean intensity = 30.9 ± 39.8 LPG; range = 0.6–119.9 LPG	[14, 23, 60]
*Trichinella nelsoni*	Encapsulated	Eastern and Southern Africa	Wild carnivorous mammals	PCR-based identification; NGS approaches	Serengeti, Tanzania: prevalence in lions = 12%; prevalence in spotted hyenas = 23%	[14, 23, 61]
*Trichinella * *patagoniensis*	Encapsulated	South America	Wild mammals, particularly pumas	Molecular identification; ITS-1 amplicon NGS	Argentina: two pumas tested positive; among documented outbreak associated sources, domestic pigs accounted for 77%, wild boars for 22%, and pumas for 1%	[14, 23, 62]
*Trichinella * *chanchalensis*	Encapsulated	North America; geographically restricted natural foci	American martens and other wild carnivores	Multiplex PCR; PCR-RFLP; NGS-based identification	Wild carnivores in Yukon, Canada: overall *Trichinella* prevalence = 74% (158/213); host-specific prevalence = 16.7%–86.4%; median intensity = 1.2–13.5 LPG; T6 intensity was 17-fold higher than that of *T. chanchalensis*	[23, 54, 63]
*Trichinella * *pseudospiralis*	Non-encapsulated	Eurasia, North America, and Australia	Birds and mammals	Molecular identification required; ITS-1 amplicon NGS	Florida panthers in Florida, USA: overall *Trichinella* prevalence = 21.4% (24/112); *T. pseudospiralis* = 14.3%; mixed *T. pseudospiralis* and *T. spiralis* infections = 1.8%	[14, 23, 64]
*Trichinella * *papuae*	Non-encapsulated	Southeast Asia and Oceania	Reptiles and mammals	PCR-based identification; NGS approaches	Papua New Guinea: prevalence in wild pigs = 11.5%; anti-*Trichinella* IgG detected in 10.0% of 1,536 humans tested	[14, 23, 65]
*Trichinella * *zimbabwensis*	Non-encapsulated	Africa	Reptiles and mammals	Molecular identification; ITS-1 amplicon NGS	Farmed Nile crocodiles in Zimbabwe: prevalence = 39.5% (256/648); wild Nile crocodiles in Mozambique = 20%; monitor lizards in Zimbabwe = 17.6%	[14, 23, 66]
Genotype T6	Encapsulated genotype	Arctic and subarctic regions of North America	Arctic foxes and other wild carnivores; mixed infections with *T. nativa* occur	Multiplex PCR; PCR-RFLP; NGS for detecting mixed infections	Wolverines in Yukon, Canada: T6 prevalence = 76%; mixed T6 and *T. nativa* infections = 12%; mean overall infection intensity = 22.6 ± 39 LPG; range = 0.1–295 LPG	[19, 54, 23, 57]
Genotype T8	Encapsulated genotype	Southern Africa; geographically restricted distribution	Large wild carnivores, including lions, hyenas, and leopards	ITS-1 amplicon NGS	Greater Kruger National Park, South Africa: lions = 4.0% (4/98); hyenas = 3.8% (1/26); leopards = 14.3% (1/7)	[35, 23, 67]
Genotype T9	Encapsulated genotype	Japan; geographically restricted natural foci	Japanese black bears, brown bears, raccoon dogs, foxes, and raccoons	ITS-1 amplicon NGS	Japan: prevalence in brown bears = 2.5% (6/236); prevalence in Japanese black bears = 0.9% (1/117)	[35, 23, 68]

ES = Excretory-secretory; IgG = Immunoglobulin G; ITS-1 = Internal transcribed spacer 1; LPG = Larvae per gram; NGS = Next-generation sequencing; PCR = Polymerase chain reaction; PCR-RFLP = Polymerase chain reaction-restriction fragment length polymorphism; USA = United States of America.

The evolutionary diversification of the genus *Trichinella* has resulted in its separation into two principal phylogenetic groups. The formation of a collagenous capsule around the nurse cell–larva complex represents a major adaptive characteristic of the encapsulated clade. This structure protects the parasite from the host immune response and facilitates its prolonged persistence in skeletal muscle tissues.

In contrast, the occurrence of non-encapsulated species, such as *T. pseudospiralis*, presents a substantial challenge for routine veterinary diagnosis. Screening procedures that rely predominantly on visualization of collagenous capsules may yield false-negative findings, thereby increasing the risk of underdetecting non-encapsulated infections during conventional trichinelloscopy. Artificial digestion is therefore considered the diagnostic gold standard for routine meat inspection because it enables the recovery of both encapsulated and non-encapsulated larvae.

**Figure 3:**
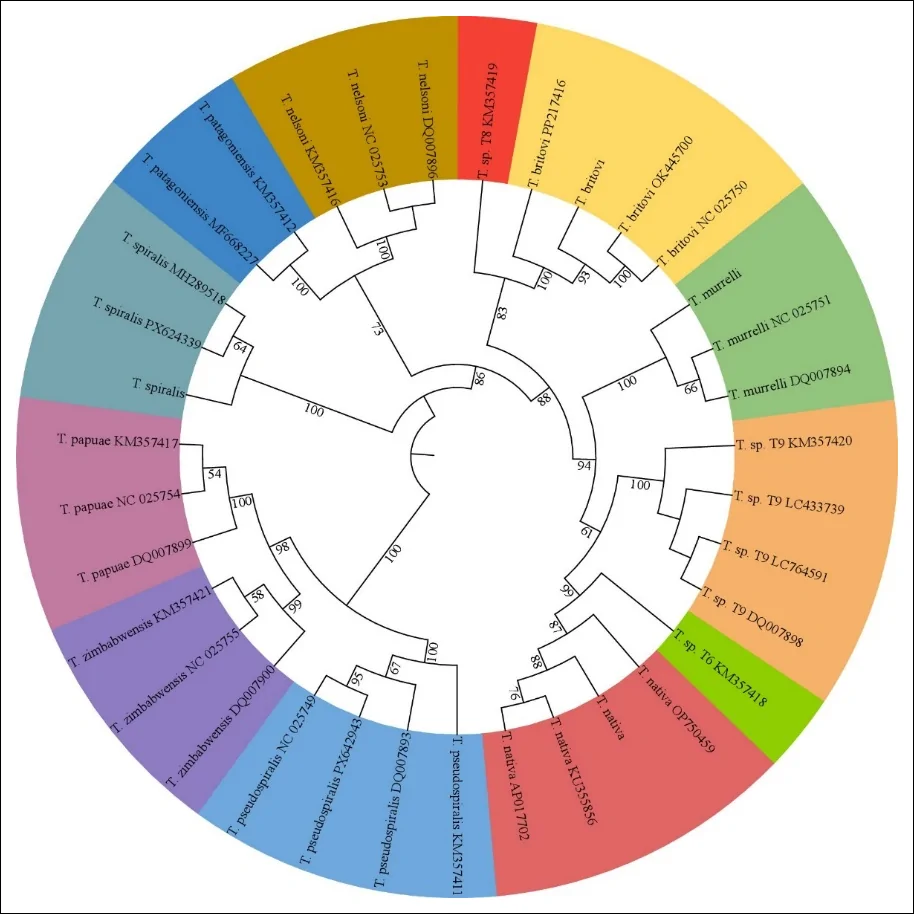
Phylogenetic relationships among recognized *Trichinella* species and genotypes T6, T8, and T9 based on nucleotide sequence comparisons. Values at the branch nodes indicate bootstrap support, and colored sectors represent individual species or genotype clusters [Source: The Figure was prepared by Nurtay Gubaidullin with the assistance of IQTree 1.6.12 and ITOL].

Nevertheless, larval recovery alone cannot reliably determine species or genotype. Molecular identification is necessary to resolve taxonomic differences that influence geographic distribution, reservoir-host associations, environmental resistance, and transmission dynamics. Phylogenetic analysis therefore provides an essential framework for distinguishing encapsulated from non-encapsulated taxa and clarifying the relationships among recognized species and unresolved genotypes. The phylogenetic tree presented in Figure 3 illustrates the genetic separation of the principal *Trichinella* taxa, including the non-encapsulated species *T. pseudospiralis*, and highlights the distinct phylogenetic positions of genotypes T6, T8, and T9.

The phylogenetic tree demonstrates clear interspecific differentiation within the genus *Trichinella*, with the recognized species and genotypes forming distinct clusters. Most species level clades, including *T. spiralis*, *T. patagoniensis*, *T. papuae*, *T. murrelli*, and genotype T9, are supported by high bootstrap values of 96%–100%, indicating strong phylogenetic stability. Other clusters, including *T. nativa*, *T. britovi*, *T. pseudospiralis*, and *T. zimbabwensis*, show moderate-to-high bootstrap support of 52%–83%, reflecting close evolutionary relationships among some taxa.

Genotypes T6, T8, and T9 form separate evolutionary lineages. Genotype T6 is positioned close to *T. nativa*, whereas genotype T8 clusters near *T. britovi* with bootstrap support of approximately 83%. Genotype T9 forms a strongly supported clade with 100% bootstrap support and is positioned near *T. murrelli*. These findings support the genetic distinctiveness of T6, T8, and T9 while indicating that additional molecular evidence is required to determine whether they should be formally recognized as separate species.

The genus *Trichinella* should therefore be regarded as a phylogenetically and ecologically heterogeneous taxonomic complex comprising recognized species and unresolved genotypes that differ in their geographic distribution, host range, environmental adaptation, zoonotic importance, and level of scientific characterization. Contemporary classification of the genus depends on the integration of morphological, biological, ecological, and molecular genetic evidence. The distinction between encapsulated and non-encapsulated taxa has not only taxonomic importance but also direct diagnostic relevance because it influences the sensitivity and reliability of routine larval detection in muscle tissues.

Diagnostic approaches developed primarily for *T. spiralis* may not provide equally reliable detection of other members of the genus because of interspecific antigenic variability, differences in seroconversion, and difficulties in recognizing mixed infections. High-resolution molecular typing is therefore particularly important for the surveillance of natural foci, the investigation of foodborne outbreaks, and the characterization of epidemiologically significant taxa such as *T. nativa* and genotypes T6–T9. Collectively, the available evidence supports the transition toward standardized, high-resolution molecular technologies as the basis for contemporary taxonomy, reliable species level diagnosis, and comparable epizootiological surveillance within the genus *Trichinella*.

**PATHOGENESIS:** A UNIVERSAL INTESTINE-TO-MUSCLE PROGRESSION

The biology of *T. nativa* generally follows the pathogenic pattern characteristic of the genus *Trichinella*; however, species-specific features may influence the clinical course and timing of diagnostic detection. Following ingestion of meat containing encapsulated first stage muscle larvae (L1), the larvae are released through gastric digestion and subsequently invade the epithelium of the small intestine, initiating the intestinal phase. Within the first 48 h, the larvae undergo four molts and reach sexual maturity. Production of newborn larvae (NBL) generally begins at 5–7 days post-infection (dpi), while adult worms may remain in the intestine for approximately 4 weeks [14].

Biologically, *T. nativa* has a lower reproductive capacity in mammalian hosts than *T. spiralis*. Whereas a single female *T. spiralis* may produce up to 1,500 NBL, a female *T. nativa* typically produces approximately 300–700 larvae. This lower larval output may be accompanied by prolonged intestinal persistence. In addition, *T. nativa* may exhibit distinct immune-evasion characteristics, including reduced expression of highly immunogenic tyvelose-containing glycans in its ES products, potentially limiting early B-cell activation and attenuating initial immunoglobulin M and immunoglobulin G responses.

### Intestinal phase and early diagnostic limitations

The intestinal phase of trichinellosis caused by *T. nativa* is generally transient and clinically nonspecific. Gastrointestinal signs, including nausea, diarrhea, and abdominal pain, may develop within 1–2 days after consumption of contaminated meat and usually persist for 2–7 days. These nonspecific manifestations complicate early clinical recognition. This period is also frequently diagnostically silent because *Trichinella*-specific antibodies may not become detectable until 3–5 weeks after infection, substantially later than the onset of gastrointestinal signs. Consequently, epidemiological information, particularly a history of consuming game meat or products not subject to veterinary inspection, is critical in the early phase [13].

**Figure 4:**
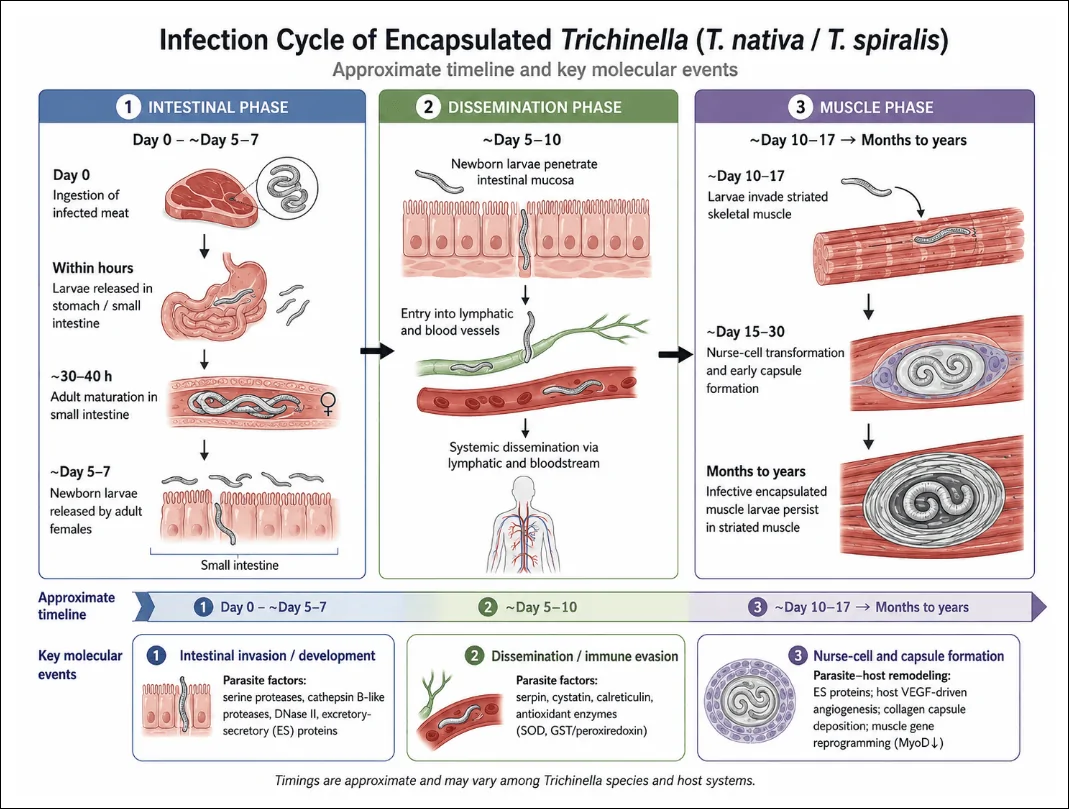
Progression of *Trichinella nativa* infection from the intestinal phase to systemic larval dissemination and muscle encystment. Following ingestion of infected meat on day 0, muscle larvae are released by gastric digestion and enter the small intestine. Larvae develop rapidly within the intestinal epithelium and mature into adult worms within approximately 30–40 h. Fertilized females begin releasing newborn larvae mainly at 5–7 dpi. These larvae penetrate the intestinal mucosa and disseminate through the lymphatic and hematogenous circulation, primarily during 5–10 dpi. During the muscular phase, larvae reach striated skeletal muscles and invade muscle fibers from approximately 10–17 dpi, initiating nurse cell transformation. Host cell remodeling and collagen capsule formation occur predominantly during 15–30 dpi. Mature encapsulated larvae may persist in skeletal muscles for months or years, maintaining the chronic muscular phase of infection. These time frames are approximate and may vary according to the *Trichinella* species, infectious dose, and host characteristics [Source: Figure prepared by Orken Akibekov with the assistance of Gemini (Google) for AI-assisted figure generation and Canva for figure design and final layout].

Following fertilization, female *T. nativa* begin larviposition. NBL are generally released from approximately day 5 onward and penetrate the intestinal mucosa before disseminating through the lymphatic and hematogenous circulation to striated skeletal muscles. This process, schematically illustrated in Figure 4, transforms a localized intestinal infection into a systemic disease.

### Larval migration and systemic manifestations

The migratory phase is associated with the development of a pronounced immune-inflammatory response, including eosinophilia and increased serum concentrations of muscle-associated enzymes such as creatine kinase. Clinical and laboratory observations indicate that eosinophilia may become evident approximately 10 days after infection [69]. Nevertheless, serological confirmation often remains difficult during this period because specific immunoglobulin G antibodies generally become detectable only 3–5 weeks after infection. Previous reviews have reported detection periods ranging from 12 to 60 dpi, depending on the infectious dose, infecting species, and individual host response, thereby explaining the risk of false-negative serological findings during early infection [13, 70]. For *T. nativa*, the reported serological detection window may extend from approximately 21 to 45 dpi, representing a delay of 7–14 days compared with the approximately 14–30 dpi window commonly described for *T. spiralis*.

During larval migration and early muscle invasion, trichinellosis typically manifests as fever, myalgia, periorbital edema, and severe weakness. Disease severity is influenced by the infectious dose, parasite species, and host immune response [71]. These manifestations have been documented in outbreaks associated with game meat. During a 2022 multistate outbreak linked to black bear meat, myalgia was reported in 83% of patients, fever in 67%, and periorbital edema in 50%, reflecting the characteristic clinical pattern of the early systemic phase [21].

Species-specific characteristics described for *T. nativa* include a less intense but potentially more prolonged intestinal phase than that associated with *T. spiralis*, which may further complicate early recognition [24]. Such interspecific differences may also have therapeutic relevance, as prolonged persistence of adult worms in the intestine could reduce the effectiveness of very brief anthelmintic regimens and warrant careful clinical assessment of treatment duration. Individuals with pre-existing gastrointestinal disease or immunosuppression may also experience more severe or prolonged intestinal manifestations.

Long-term clinical outcomes may be influenced by the persistence of larvae in skeletal muscles. Because *T. nativa* larvae can remain viable for prolonged periods, patients may experience persistent myalgia, fatigue, and muscle remodeling after the acute phase. Outbreaks associated with bear meat are especially informative because they demonstrate the relationship among epidemiological history, clinical presentation, and species-specific biological characteristics. In northern Saskatchewan, trichinellosis was diagnosed based on clinical manifestations, complete blood count findings, elevated creatine kinase concentrations, serological results, and a history of consuming contaminated bear meat. Similarly, during an outbreak in Northern Ontario, affected patients developed severe systemic manifestations after an initial intestinal phase. These observations demonstrate the importance of integrating clinical findings with exposure history and species level parasite identification when evaluating prognosis and identifying the source of infection [72, 73].

### Muscular phase and nurse cell formation

The formation of the nurse cell, a specialized larva–muscle fiber complex, is a central event in the pathogenesis of trichinellosis. After penetrating a myocyte, the larva induces extensive structural and metabolic remodeling of the host cell. Maturation of this complex generally requires approximately 15–20 days [74–76]. In *T. nativa*, structural maturation of the nurse cell complex and completion of collagen capsule formation may occur over approximately 20–28 days, compared with the more restricted interval of approximately 15–20 days described for *T. spiralis*.

As in other encapsulated species, prolonged persistence of *T. nativa* larvae in skeletal muscles may result in sustained antigenic stimulation. However, early serological diagnosis remains challenging because specific antibodies generally become detectable only 3–5 weeks after infection and may appear even later in low intensity infections. Consequently, negative serological findings obtained during the early stages of disease cannot reliably exclude infection and should be interpreted cautiously [70, 77, 78].

The exceptional cold tolerance of *T. nativa* further increases its epidemiological importance because larvae may remain infective after conventional freezing of game meat [21]. Interspecific antigenic variability creates an additional diagnostic challenge. Antigens that are strongly expressed and immunoreactive during early *T. spiralis* infection and that form the basis of several widely used serological assays may not elicit equivalent responses during *T. nativa* infection. Direct extrapolation of assays developed for *T. spiralis* may therefore result in reduced or inconsistent sensitivity and specificity for *T. nativa* [23]. These limitations emphasize the need for high-resolution species identification and the development and validation of species-specific antigens.

### Phase-dependent diagnostic implications

The life cycle of *Trichinella* follows an intestine-to-muscle progression in which the reliability of laboratory confirmation depends strongly on the stage of infection. During the early intestinal phase, larvae mature into adults and gastrointestinal signs may occur before specific antibodies become detectable. From approximately day 5 onward, NBL disseminate through the lymphatic and hematogenous circulation. Muscle invasion and nurse cell formation then become evident from approximately 15–20 dpi.

A critical diagnostic interval therefore exists between the onset of systemic manifestations, including fever and myalgia, and reliable serological confirmation. This interval typically lasts 3–5 weeks and may be especially prolonged in *T. nativa* infection. The comparatively delayed antibody response may render conventional serological assays insensitive during the period when early therapeutic intervention is most important. Accordingly, a negative early serological result should not rule out trichinellosis when clinical and epidemiological evidence strongly suggests infection.

### Clinical and diagnostic significance

*T. nativa* is an epidemiologically and diagnostically important species in which the general pathogenic progression of trichinellosis is combined with distinctive biological characteristics of practical relevance. Its comparatively prolonged intestinal phase, delayed serological response, extended survival in skeletal muscle, antigenic variability, and exceptional cold tolerance collectively complicate early diagnosis and increase the risk associated with frozen game meat. Clinical assessment should therefore integrate exposure history, gastrointestinal and systemic manifestations, eosinophil counts, muscle enzyme concentrations, repeated serological testing, and molecular species identification whenever suitable material is available.

The available evidence supports the use of species-adapted approaches for the diagnosis and epidemiological control of *T. nativa*. Further research should prioritize the development of sensitive early-stage biomarkers, standardized, species-specific recombinant antigens, and reproducible molecular assays that can differentiate *T. nativa* from closely related taxa and detect mixed infections.

**FROM CONVENTIONAL METHODS TO NGS:** DIAGNOSIS OF TRICHINELLOSIS

### Artificial digestion

Artificial digestion of muscle tissue remains the principal direct method for detecting *Trichinella* larvae across the genus [79]. For *T. nativa*, this method remains the diagnostic gold standard for food safety inspection, epizootiological surveillance, and laboratory confirmation of infection. In addition to detecting muscle-stage larvae, artificial digestion provides biological material for subsequent molecular species identification [80].

To ensure standardization and reproducibility, the procedure is performed in accordance with validated recommendations issued by the World Organization for Animal Health and the International Commission on Trichinellosis [81]. In the standard protocol, 100 g of minced muscle tissue is digested in 2 L of tap water maintained at 46°C ± 2°C. The digestion fluid contains hydrochloric acid at a final concentration of 1% (v/v), corresponding to approximately 25 mL of 25% hydrochloric acid or 16 mL of 37% hydrochloric acid, and pepsin powder at 0.5% (w/v), equivalent to 10 g of pepsin with an enzymatic activity of 1:10,000 NF or 1:2,000 FIP.

**Figure 5:**
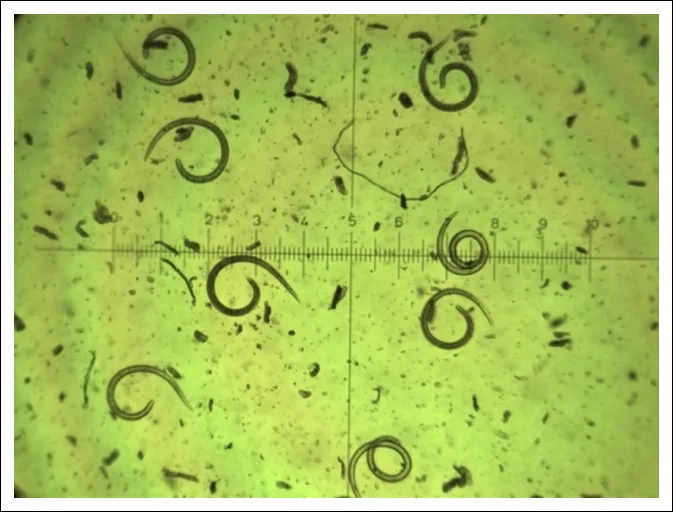
Recovery and microscopic examination of viable *Trichinella* larvae from infected meat. (A) Isolation of live larvae using the Baermann technique. Minced muscle tissue was placed in a funnel containing warm water, allowing motile larvae to migrate downward and accumulate in the lower portion of the apparatus for collection. (B) Light microscopic examination of purified viable larvae following sedimentation and concentration. Intact larvae are visible after the removal of tissue debris and other contaminants. Microscopic examination was performed at 100× magnification using an ocular micrometer [10].

The sample is digested under magnetic stirring for 30–45 min or until complete tissue dissolution. The digest is then passed through a 180-µm brass or stainless-steel sieve into a glass sedimentation funnel and allowed to settle for 30 min. Approximately 40 mL of sediment is collected and transferred to a smaller conical cylinder for a second 10-min sedimentation period. The supernatant is subsequently removed, leaving approximately 10 mL of concentrated sediment for systematic microscopic examination in a gridded Petri dish to identify liberated larvae (Figure 5).

In wildlife surveillance programs in Kazakhstan, the standard protocol has been adapted by increasing the muscle sample mass to 25–50 g per carcass and prioritizing tissues from recognized predilection sites. These adaptations are intended to improve detection in wild carnivores, in which infections may occur at low intensity and larvae may be distributed unevenly among muscle groups.

**Figure 6:**
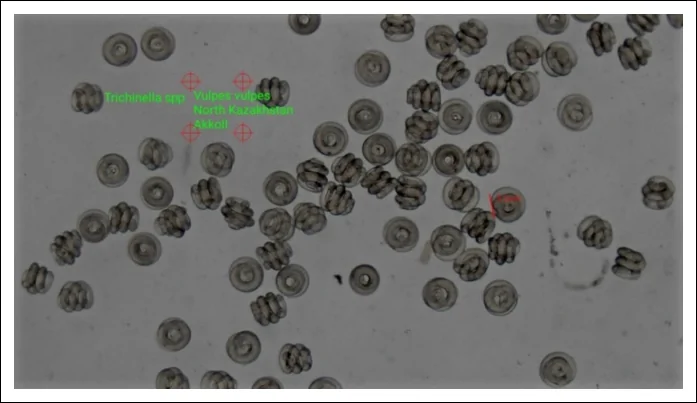
*Trichinella* spp. muscle-stage larvae recovered from red fox (*Vulpes vulpes*) muscle tissue collected in Akkol, Northern Kazakhstan. Larvae were isolated by artificial digestion, followed by sedimentation, concentration, and light microscopic examination. The characteristic coiled morphology is consistent with viable muscle-stage *Trichinella* larvae. Species level identification was subsequently confirmed by molecular analysis [10].

Artificial digestion has been applied in both its conventional acid–pepsin form and through several methodological modifications. These include increasing the sample mass, selecting predilection muscles, pooling samples, and integrating subsequent molecular typing [82, 83]. However, several practical limitations are particularly relevant to the detection and morphological identification of *T. nativa* larvae (Figure 6).

In Arctic and subarctic sylvatic cycles, infection is frequently characterized by low larval intensity and a markedly heterogeneous distribution of larvae among muscle groups. Consequently, a negative finding from a small sample or from tissue collected outside a recognized predilection site does not definitively exclude epidemiologically relevant contamination [84]. This limitation is particularly important for *T. nativa* because even low numbers of viable larvae in wildlife meat may remain infective after prolonged freezing. Failure to detect low intensity infection may therefore have greater public health consequences than in controlled swine-production systems predominantly associated with *T. spiralis*.

Current international guidance emphasizes that the sensitivity of artificial digestion depends primarily on the mass of tissue examined and the selection of an appropriate predilection site [84]. Field evaluations indicate that the method has very high diagnostic specificity because detection is based on direct visualization of the parasite. However, field sensitivity varies substantially with infection intensity, sample mass, tissue selection, and infection stage. Sensitivity may approach 95.0%–98.5% in heavily infected wildlife hosts but may decline to <65.0% in low intensity or early sylvatic infections in which larval density is minimal.

Standard food safety protocols are generally designed to detect animals carrying at least 1 larva per gram (LPG), whereas wildlife surveillance and epidemiological research often require greater analytical sensitivity [85]. When samples are collected from predilection muscles, examining 100 g of tissue may yield an estimated detection limit of approximately 0.01 LPG. This level of sensitivity is particularly important for *T. nativa*, for which routine examination of only 1–5 g of tissue per animal may underestimate the true occurrence of infection.

Artificial digestion has therefore been adapted in northern surveillance programs to address public health and field-specific requirements. Studies of walrus meat showed that the tongue was the most informative tissue for detecting *Trichinella*, with larval burdens approximately 2–6 times higher than those recorded in the pectoral and intercostal muscles. On this basis, examination of at least 10 g of tongue tissue was recommended to improve diagnostic reliability [81]. This sampling strategy was subsequently incorporated into the Nunavik Trichinellosis Prevention Program [82], in which pooled tongue samples from harvested walruses are examined by artificial digestion. Decisions regarding the suitability of the meat for human consumption are then based on the screening results.

An additional limitation of artificial digestion is its inability to identify larvae reliably at the species level based on morphology alone. Although the method confirms the presence of *Trichinella* spp., it cannot definitively distinguish *T. nativa* from other species or genotypes without subsequent molecular characterization [79]. Contemporary diagnostic practice therefore increasingly considers artificial digestion as the first stage of a two-step workflow comprising larval recovery followed by PCR-based typing or sequencing.

This combined approach has been used in studies of Alaskan mammals, in which larvae recovered through artificial digestion were genotyped to differentiate among northern *Trichinella* taxa [86]. It is especially valuable in regions where closely related taxa, mixed infections, or geographically overlapping transmission cycles occur.

A similar diagnostic framework has been implemented in Kazakhstan. During a 10-year surveillance study of wild carnivores, larvae were first recovered by artificial digestion and subsequently analyzed by PCR targeting the *5S rDNA* intergenic spacer region, followed by nucleotide sequencing. This strategy enabled positive findings to be interpreted not merely as nonspecific *Trichinella* spp. infections but as evidence of the circulation of *T. nativa* among wild carnivore populations [10].

Artificial digestion should therefore be regarded as an indispensable but incomplete diagnostic stage for *T. nativa*. Its effectiveness is maximized by examining an adequate tissue mass, selecting recognized predilection muscles, and subjecting recovered larvae to molecular confirmation. This integrated strategy accounts for the low intensity and heterogeneous distribution of sylvatic infections while addressing the substantial epidemiological importance of even small numbers of freeze-resistant larvae in wild game meat.

### ES antigens and the serological window

Serological diagnosis of *T. nativa*, as with trichinellosis in general, is constrained by the temporal disconnect between parasite development and the onset of a detectable humoral immune response [87]. ES antigens derived from muscle larvae (ML ES) remain the most widely used antigenic preparations and are recommended as the reference antigens for enzyme-linked immunosorbent assay (ELISA) and immunoblotting. However, their principal limitation is their relatively low sensitivity during the early stages of infection [88]. Recent reviews emphasize that validated early diagnostic assays based on well-characterized antigens remain limited. Consequently, serological findings should always be interpreted together with the patient's clinical presentation and epidemiological history [70, 89].

**The serological window:** The limitations of conventional ML ES antigens are most evident during the so-called serological window. According to the International Commission on Trichinellosis guidelines, seroconversion in humans generally occurs between the third and fifth weeks after infection. Specific immunoglobulin G antibodies become detectable approximately 12–60 days after exposure, depending on the infective dose, *Trichinella* species, and individual host immune response. Therefore, a negative ELISA result obtained during the early phase of infection does not exclude trichinellosis and should not be considered sufficient to rule out the disease when compatible epidemiological exposure has been documented [13, 90].

Because of this limitation, considerable research has focused on identifying antigens expressed during the earliest developmental stages of the parasite, particularly those derived from intestinal larvae, adult worms, and NBL. Reviews of serodiagnostic strategies indicate that stage-specific antigens can detect antibody responses earlier than conventional ML ES preparations because the immunodominant epitopes expressed during the intestinal phase are either absent or poorly represented in muscle larval ES antigens [70, 89].

**Recombinant antigens for early diagnosis:** The potential advantages of stage-specific recombinant antigens have been demonstrated experimentally using *T. spiralis*. Hu *et al.* [12] reported that recombinant elastase-1 (rTsEla) enabled substantially earlier antibody detection than conventional ML ES antigen. In an experimental mouse model, rTsEla-based ELISA detected specific immunoglobulin G antibodies as early as 10–12 days post-infection (dpi) and achieved 100% diagnostic sensitivity by 14 dpi. In contrast, ELISA using the conventional ML ES antigen showed delayed antibody detection and lower sensitivity during early infection. Based on these findings, the authors proposed rTsEla as a promising alternative antigen for early serological diagnosis.

For *T. nativa*, however, direct extrapolation of antigenic platforms validated for *T. spiralis* remains methodologically challenging. Although sufficient antigenic conservation exists within the genus to permit cross-species recognition in conventional serological assays, interspecific differences in antigen composition and immunoreactivity may substantially influence the sensitivity of early-stage testing and complicate interpretation at the species level. This issue is particularly important for *T. nativa* because definitive species identification directly affects the epidemiological assessment of residual infection risk associated with frozen meat [12, 70].

**Figure 7:**
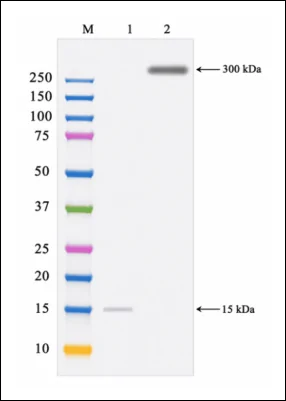
Immunoblot analysis of *T. spiralis* antigenic fractions using positive reference serum. Lane M: Prestained protein molecular-weight marker with bands at 10, 15, 20, 25, 37, 50, 75, 100, 150, and 250 kDa. Lane 1: ES antigen demonstrating a highly immunoreactive band at approximately 15 kDa. Lane 2: Somatic antigen demonstrating a prominent high-molecular-weight immunoreactive band at approximately 300 kDa. Negative reference sera obtained from noninfected animals showed no specific immunoreactive bands, confirming the diagnostic specificity of the identified antigenic fractions [91].

Akibekov *et al.* [91] isolated both ES and somatic antigens from *T. spiralis* and demonstrated their diagnostic performance in ELISA and immunoblot assays (Figure 7).

**Species-specific antigens for T. nativa:** At present, only a limited number of serodiagnostic antigens have been characterized specifically for *T. nativa*. Akibekov *et al.* [24] demonstrated that the intestinal phase of *T. nativa* persists longer than that of *T. spiralis*, with serine protease gene expression remaining detectable until approximately 30 dpi. These findings suggest that serine protease may represent a promising candidate antigen for early diagnosis. Nevertheless, the authors emphasized that available information regarding *T. nativa* serine protease remains insufficient and that further studies are required to evaluate its species specificity and antigenic properties.

More recently, comparative investigations of recombinant serine proteases from *T. spiralis* and *T. nativa* demonstrated encouraging results for the recombinant *T. nativa* antigen rTnsp-4E. Under experimental conditions, this recombinant protein enabled antibody detection as early as 7 dpi. Diagnostic evaluation demonstrated a sensitivity of 96.8%, specificity of 98.1%, positive predictive value of 95.5%, and negative predictive value of 98.7%, substantially outperforming conventional crude ES antigens during the pre-encystment phase [25].

Evaluation of cross-reactivity showed no detectable reactions with sera from animals infected with unrelated helminths, including *Echinococcus granulosus*, *Toxocara* *canis*, and *Ascaris suum*. Limited intragenus cross-reactivity was observed with *T. britovi* (8.2%) and *T. spiralis* (4.5%), indicating that additional epitope refinement may further improve species specificity. Although these findings require validation using larger independent cohorts and naturally infected field populations, they provide strong experimental evidence that *T. nativa* requires dedicated antigenic platforms rather than direct adaptation of diagnostic systems originally developed for *T. spiralis* [25].

**Emerging antigenic platforms:** Another promising approach involves the development of monoclonal antibodies directed against ML ES antigens. Akibekov *et al.* [92] generated monoclonal antibodies against *T. spiralis* ES antigens that recognized a 75-kDa protein by Western blot analysis. The authors proposed that this immunoreactive protein could serve as the basis for developing more specific serological assays. However, because these studies were performed exclusively with *T. spiralis* and have not been independently validated for *T. nativa*, their implications for species-specific diagnosis remain preliminary.

Additional antigenic platforms currently under investigation include preparations derived from adult worms, intestinal infective larvae, and NBL, as well as purified or recombinant TSL-1 and tyvelose-containing glycoproteins. Nevertheless, available reviews consistently indicate that most experimental evidence has been generated using *T. spiralis* or highly controlled laboratory models. Standardized, fully validated, and interlaboratory reproducible early serological assays applicable across *Trichinella* species, including *T. nativa*, remain unavailable [70, 93].

A further promising direction involves identifying novel antigenic targets through molecular and bioinformatic analyses. Zhumalin *et al.* [94] evaluated the immunogenic potential of the *T. nativa* fatty acid transport protein 1 (FATP1) and PX-domain proteins using BepiPred 3.0. Computational prediction identified several potential B-cell epitopes, suggesting that these proteins may represent suitable candidates for future diagnostic assay development. However, these findings are based primarily on in silico analyses and require comprehensive experimental and clinical validation.

**Future perspectives:** Despite their widespread use, ML ES antigens do not overcome the fundamental limitation of early serological diagnosis. The prolonged serological window, interspecific antigenic variability, and the limited availability of validated *T. nativa*-specific reagents continue to restrict the diagnostic performance of conventional assays.

Current evidence therefore supports a dual diagnostic strategy. The first priority is the development and independent validation of recombinant antigens specifically optimized for *T. nativa*. The second is the routine integration of serological testing with molecular species identification and epidemiological investigations, particularly during outbreak investigations and surveillance of Arctic and boreal sylvatic transmission cycles. Such an integrated approach offers the greatest potential to improve the sensitivity, specificity, and epidemiological interpretation of serological diagnosis of *T. nativa*.

**MODERN IDENTIFICATION APPROACHES:** PCR MARKER SCHEMES, SEQUENCING, AND NGS

Modern identification of *T. nativa* increasingly depends on molecular methods because detection of *Trichinella* spp. larvae in muscle tissue alone is insufficient for species level interpretation. Confirmation of this cold-adapted taxon is epidemiologically important because it influences assessment of the risk of human infection, recommendations regarding meat storage and processing, and classification of transmission within Arctic and subarctic natural cycles [4, 19, 21]. Consequently, recent research has shifted from descriptive epizootiological surveillance to high-resolution species-identification frameworks. These approaches are specifically intended for complex wildlife samples, which are frequently characterized by low larval burdens, heterogeneous larval distribution among muscles, tissue degradation, and mixed infections involving multiple *Trichinella* taxa [4, 23].

### Two-stage diagnostic framework

In practice, this development has resulted in a two-stage diagnostic algorithm that is now widely applied in field investigations: recovery of larvae by artificial digestion followed by molecular species confirmation. This approach is consistent with the International Commission on Trichinellosis guidelines, which emphasize that muscle larvae of different *Trichinella* taxa are morphologically indistinguishable and therefore require genotyping for definitive identification [43].

The practical value of this framework was demonstrated during a 10-year surveillance study of wild carnivores in Kazakhstan. Larvae recovered by artificial digestion were analyzed by PCR targeting the *5S rDNA* intergenic spacer, followed by confirmatory Sanger sequencing [10]. PCR amplification was performed using the following primers:

F = 5′-GCGAATTCTTGGATCGGAGACGGCCTG-3′ R = 5′-GCTCTAGACGAGATGTCGTGTGTTTCAACG-3′

The thermal cycling conditions comprised an initial denaturation at 95°C for 5 min, followed by 35 cycles of denaturation at 94°C for 30 s, primer annealing at 55°C for 45 s, and extension at 72°C for 1 min. A final extension was performed at 72°C for 10 min. The assay generated a distinct 127-bp amplicon characteristic of *T. nativa*. This combined approach enabled positive findings in wolves, foxes, and badgers to be interpreted as evidence of *T. nativa* circulation rather than as nonspecific infection with *Trichinella* spp. [10].

### Multiplex PCR for primary species identification

**Figure 8:**
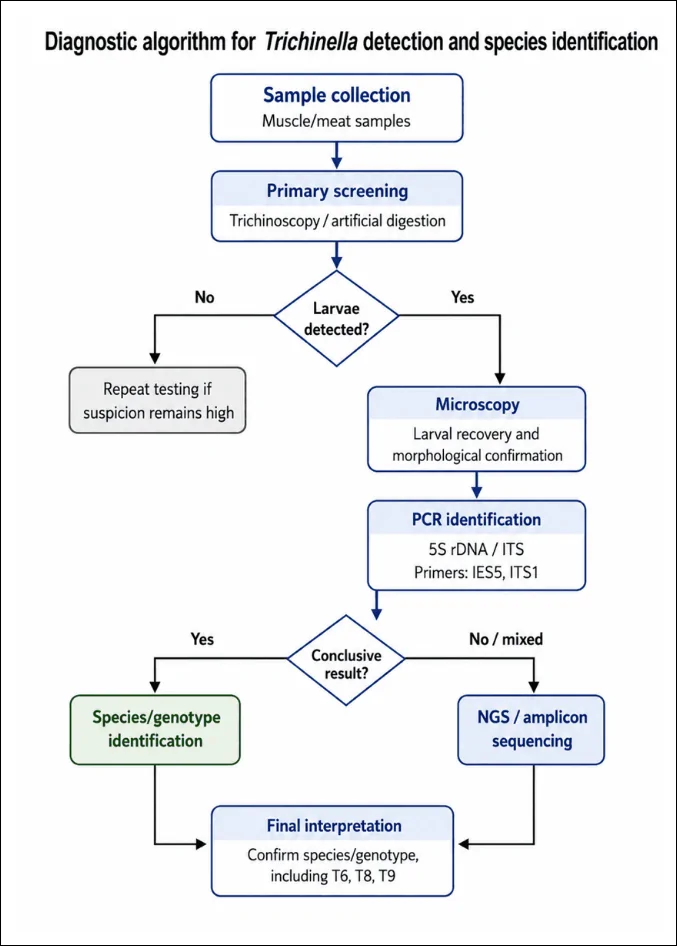
Diagnostic algorithm for the detection of *Trichinella* larvae and molecular identification of species and genotypes. Initial screening is performed using trichinoscopy and/or artificial digestion of muscle or meat samples. When larvae are detected, they are recovered and examined microscopically. Molecular identification is subsequently conducted using PCR assays targeting the *5S rDNA* intergenic spacer and internal transcribed spacer regions with IES5- and ITS1-based primer systems. Samples that produce inconclusive amplification patterns or indicate possible mixed infection are subjected to NGS-based amplicon sequencing. The final interpretation confirms the species or genotype, including epidemiologically important genotypes T6, T8, and T9 [Source: Figure prepared by Saulet Issayev with the assistance of Gemini (Google) for AI-assisted figure generation and Canva for figure design and final layout].

Multiplex PCR targeting ribosomal DNA regions remains the principal molecular method for routine differentiation of *Trichinella* species and genotypes (schematically illustrated in Figure 8). Its main advantages include compatibility with internationally recognized reference protocols and the ability to differentiate most accepted taxa according to characteristic amplicon profiles [43].

Despite its practical value, multiplex PCR has several limitations. Additional amplification bands, atypical amplicon profiles, low DNA concentrations, and analysis of pooled larvae may complicate interpretation. In interlaboratory studies, such factors have led to incorrect species assignments, including the misidentification of *T. murrelli* [95]. This limitation is particularly relevant to *T. nativa* surveillance because wildlife studies frequently analyze pooled larvae rather than individual specimens. Detection of an additional taxon in such pools may indicate a genuine mixed infection, laboratory contamination, or nonspecific amplification.

### Confirmatory Sanger sequencing

Sanger sequencing remains an important confirmatory and arbitration method following PCR amplification of the *5S rDNA* spacer, ITS regions, or mitochondrial markers. Its principal strengths are high sequence specificity and the ability to verify taxonomic assignment through phylogenetic analysis. These characteristics are essential when documenting the persistence or possible emergence of *T. nativa* outside its recognized geographic range and when expanding regional sequence databases for comparative population studies [10, 43].

However, Sanger sequencing has limited ability to identify minor sequence components in mixed samples. When one taxon occurs at a substantially lower abundance than another, its sequence signal may be masked within the composite chromatogram. Consequently, Sanger sequencing remains highly valuable for confirmation of single-species infections but may fail to resolve low-frequency taxa or complex mixed infections [96].

### NGS for mixed infections and low-abundance taxa

NGS has emerged as one of the most important developments in the molecular identification of *Trichinella*. Its major advantage is the ability to detect underrepresented genotypes in mixed natural infections and to distinguish low-frequency taxonomic signals from background amplification noise [23, 97]. This capability is particularly relevant to *T. nativa* because multiple cold-adapted taxa, especially *T. nativa* and genotype T6, may occur simultaneously in the same host in northern ecosystems.

The amplicon-based NGS method targeting the ITS-1 region proposed by Lobanov *et al.* [23] was designed as a broadly applicable genotyping assay for recognized *Trichinella* species and genotypes. The workflow involves targeted PCR amplification, preparation of indexed amplicon libraries, and high-throughput sequencing, commonly using an Illumina MiSeq platform with paired-end sequencing chemistry. For complex larval pools, an average sequencing depth of approximately 50,000–100,000 raw reads per sample may be used to improve detection of low-abundance sequence variants.

Downstream bioinformatic processing includes quality trimming, removal of low-quality reads, sequence denoising, and generation of amplicon sequence variants using analytical platforms such as DADA2 or QIIME 2. Taxonomic assignment is then conducted by comparing the resulting sequences with verified reference databases using BLASTn. This approach demonstrated that a single *T. nativa* larva present within a pool of 100 larvae could generate a detectable and interpretable sequence signal. Such low-abundance infections remain difficult to identify using conventional multiplex PCR or Sanger sequencing.

### Application of NGS in Arctic wildlife surveillance

The practical value of NGS has been demonstrated in recent studies of Arctic wildlife. In a 2025 investigation from Alaska, larvae recovered through artificial digestion were characterized using an amplicon-based NGS workflow on an Illumina platform. Deep sequencing and bioinformatic analysis optimized for low-frequency sequence detection revealed multiple mixed infections involving *T. nativa* and genotype T6. The study also documented a triple infection involving *T. nativa*, T6, and *T. chanchalensis* [98].

These findings indicate that NGS does more than improve the accuracy of species identification. It can substantially alter the understanding of parasite co-circulation and transmission dynamics within northern natural foci. Similarly, a Yukon study using metabarcoding and stringent sequence-quality filtering demonstrated a broader host range for *T. chanchalensis* and provided no evidence of strong interspecific competition between *T. nativa* and T6 in naturally co-infected hosts [63]. These observations suggest that the assumption of a single host carrying only a single *Trichinella* taxon is often invalid in Arctic and boreal ecosystems.

### Emerging molecular technologies

A broader range of molecular technologies is also being investigated for *Trichinella* detection and identification. In addition to conventional PCR, multiplex PCR, Sanger sequencing, and NGS, recent reviews describe the development of quantitative PCR, loop-mediated isothermal amplification, recombinase polymerase amplification, and CRISPR-Cas-based assays [97]. These technologies are particularly promising for the analysis of low intensity infections, individual larvae, and minimally processed field samples.

Although these methods have not yet replaced established international protocols, they represent an important direction for future diagnostic development. In this emerging framework, analytical sensitivity, rapid turnaround, portability, and compatibility with complex field samples are becoming as important as species specificity. However, field implementation must balance diagnostic performance against cost, laboratory infrastructure, personnel training, and access to specialized equipment.

NGS and other high-resolution methods remain most suitable for reference laboratories, confirmatory investigations, and research applications due to their cost and computational requirements. In contrast, artificial digestion and conventional multiplex PCR remain more practical for routine large-scale surveillance in regional laboratories. The most appropriate diagnostic strategy therefore depends on the epidemiological objective, sample type, expected infection intensity, and available laboratory capacity.

### Integrated molecular identification algorithm

Modern identification of *T. nativa* is increasingly based on a multitiered diagnostic framework. Artificial digestion enables larval recovery and estimation of infection intensity; multiplex PCR provides initial species or genotype screening; Sanger sequencing confirms ambiguous results and supports phylogenetic comparison; and NGS resolves the most complex samples, including mixed infections, low-abundance taxa, and suspected rare northern genotypes [10, 23, 43, 97].

For Kazakhstan and neighboring territories, this framework has both taxonomic and public health significance. Accurate species level identification of *T. nativa* is essential for evaluating the risks associated with wild game meat, interpreting the significance of freezing resistance, characterizing natural transmission foci, and developing evidence-based recommendations for meat inspection and consumer protection. Consequently, high-resolution molecular confirmation should be considered an integral component of contemporary surveillance whenever *T. nativa* or closely related northern taxa are suspected.

### Methodological evolution in the diagnostic strategy for *T. nativa*

Developing effective diagnostic systems for *T. nativa* requires a multilevel methodological framework integrating epizootiological surveillance, clinical pathology, antigen characterization, and molecular candidate selection. This comprehensive approach is necessitated by the biological characteristics of *T. nativa*, particularly its cold tolerance and sylvatic transmission pattern. Accurate differentiation from other *Trichinella* spp. is essential for evaluating food safety risks associated with natural foci and the consumption of wild game meat.

### Epizootiological surveillance as the methodological foundation

**Figure 9:**
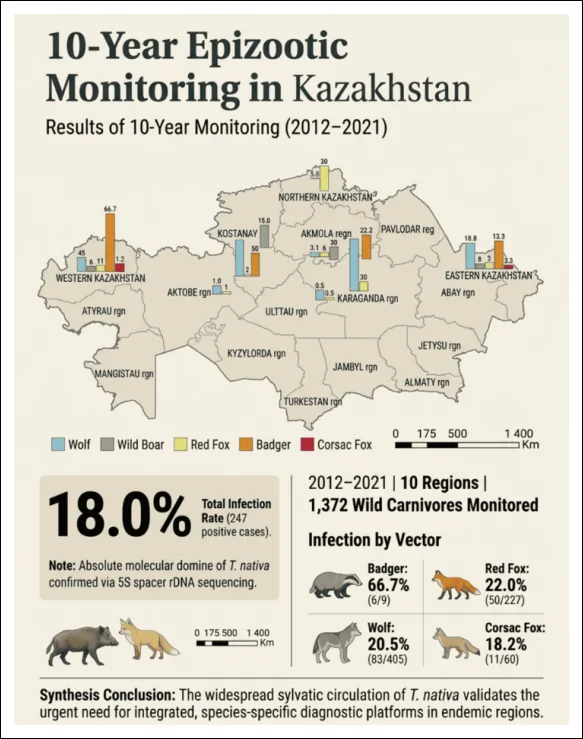
Geographic distribution of trichinellosis among five wildlife species during 10 years of surveillance in Kazakhstan [10]. The map shows the regional distribution of trichinellosis detected during epizootiological monitoring conducted from 2012 to 2021. A total of 1,372 wild animals from 10 regions were examined, of which 247 were positive, corresponding to an overall prevalence of 18.0%. Colored bars indicate infection rates among wolves, wild boars, red foxes, badgers, and corsac foxes. The highest prevalence was recorded in badgers at 66.7% (6/9), followed by red foxes at 22.0% (50/227), wolves at 20.5% (83/405), and corsac foxes at 18.2% (11/60). Molecular confirmation based on sequencing of the *5S rDNA* spacer indicated the predominance of *T. nativa*, supporting its widespread sylvatic circulation in Kazakhstan. The scale bar indicates distance in kilometers.

The methodological framework begins with long-term epizootiological monitoring to establish the natural circulation, geographic distribution, and host range of the parasite. In Central Asia, particularly Kazakhstan, a 10-year surveillance program combining artificial digestion of muscle tissues, PCR targeting the *5S rDNA* intergenic spacer, and confirmatory sequencing demonstrated the active circulation of *T. nativa* among wild carnivores, including wolves, foxes, and badgers (Figure 9) [10].

Methodologically, these findings demonstrate that positive field detections should not be reported solely as infection with an unspecified *Trichinella* spp. complex. Instead, molecular confirmation is required to identify the taxon and determine its independent epidemiological significance. Long-term surveillance also reveals substantial interspecific and interhost variability in prevalence and infection intensity. Such variation directly influences diagnostic sensitivity and increases the risk of false-negative findings in hosts carrying low larval burdens.

### Development of antigen-based diagnostic platforms

Characterization of the antigenic profile of *Trichinella* represents another major stage in the development of serological assays. Initial methodological models focused on the isolation and evaluation of ES and somatic antigens from *T. spiralis*. These investigations identified highly immunoreactive fractions, including an approximately 15-kDa ES antigen and a 300-kDa somatic antigen, using ELISA and immunoblotting [91].

Although this work was conducted using *T. spiralis*, it provided an important methodological foundation for subsequent diagnostic research. In particular, it facilitated the transition from broad antigen characterization toward the targeted selection of molecules with potential immunodiagnostic value. This research direction was subsequently expanded by generating monoclonal antibodies against ES antigens derived from *T. spiralis* muscle larvae. In a focused study, monoclonal antibodies recognized a 75-kDa protein by Western blot analysis, suggesting its potential for the development of more specific serological assays [92].

Nevertheless, comparative studies indicate that serological platforms optimized for *T. spiralis* cannot be directly extrapolated to *T. nativa* because of interspecific differences in antigen expression and immunoreactivity. These limitations support the development and validation of species-specific antigenic alternatives.

### Clinical-pathological markers of early infection

Experimental models have also been used to characterize early laboratory differences between *T. nativa* and *T. spiralis*. In *T. nativa* infection, measurable changes may occur as early as 7 days dpi, including alterations in erythrocyte- and platelet-associated parameters and changes in neutrophil and eosinophil profiles. Among the biochemical indicators assessed, creatine kinase-N-acetyl-cysteine was identified as a potentially sensitive early enzymatic marker.

At the same time, enzyme-linked immunosorbent assays using ES and somatic antigens detected antibodies from 7 dpi onward. However, the low antibody titers observed during this period confirmed the persistence of a diagnostically important serological window in the early enteral phase [69]. Methodologically, these findings demonstrate that early diagnosis of *T. nativa* should not rely on a single serological assay. Instead, hematological, biochemical, serological, and molecular indicators should be interpreted collectively.

### Serine protease as an early molecular target

Evaluation of serine protease expression in *T. spiralis* has highlighted its potential as an early diagnostic marker. Experimental findings showed that serine protease transcripts were detectable in BALB/c mice at 7 and 14 dpi in 83% of infected animals. Increasing the infective dose from 100 to 250 larvae did not significantly improve the detection rate. These findings support the use of serine protease as a candidate target for recombinant protein-based diagnostic assays [99].

This strategy is methodologically important because it supports a transition from conventional muscle larval ES antigens toward stage-associated molecular markers expressed during early infection. Such markers may improve diagnostic sensitivity before the development of a strong antibody response against muscle-stage antigens.

Comparative studies of *T. spiralis* and *T. nativa* further demonstrate that serine protease gene transcripts may serve as molecular indicators of the early enteral phase. In *T. nativa*, the enteral stage persisted from approximately 7 to 30 dpi, whereas *T. spiralis* showed earlier and more restricted developmental kinetics [24]. These temporal differences support serine protease as a stage-specific target that may bridge early molecular detection with the selection of recombinant antigens for immunological assays.

### Expansion of species-specific candidate targets

To broaden the repertoire of potential diagnostic markers, additional *T. nativa* genes have been evaluated as candidates for the development of species-specific assays. In a bioinformatic study, the *fatp1* and *px-domain* genes were analyzed for predicted B-cell epitopes, thereby identifying novel candidate antigenic regions [94].

Methodologically, this work demonstrates that the search for diagnostic targets in *T. nativa* should not be restricted to traditional ES antigens or serine proteases. Instead, a broader candidate selection strategy is required, incorporating comparative genomics, protein domain analysis, epitope prediction, species specificity, and potential for cross-reactivity. However, because these findings were derived primarily from computational analyses, experimental confirmation of protein expression, immunoreactivity, diagnostic sensitivity, and specificity remains necessary.

### Translation of candidate selection into recombinant antigen evaluation

**Figure 10:**
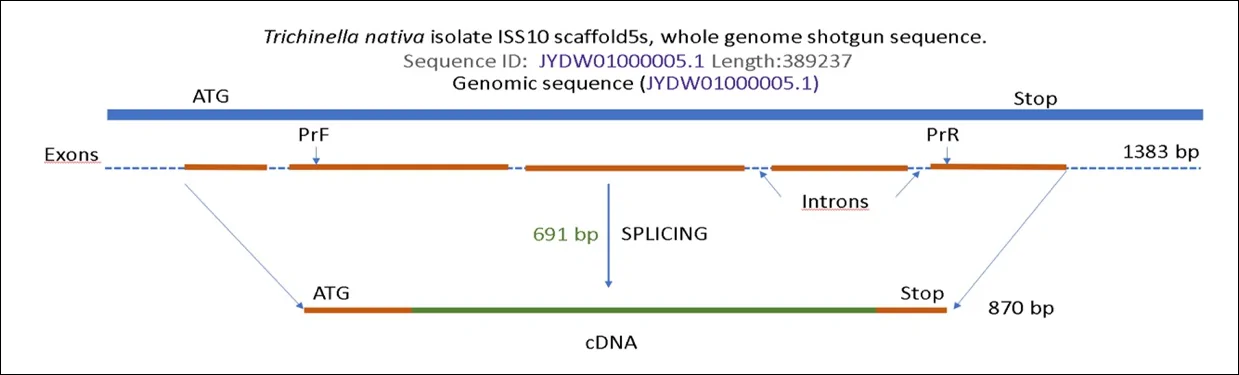
Schematic organization of serine protease gene fragments in *T. spiralis* and *T. nativa*. (A) *T. spiralis* strain ISS 195, chromosome 1, whole-genome shotgun sequence ABIR03006837.1. (B) *T. nativa* isolate ISS10, scaffold 5, whole-genome shotgun sequence JYDW01000005.1. Orange lines indicate exon regions, dashed lines indicate introns, and green segments indicate the target exon regions selected for analysis. ATG and stop indicate the boundaries of the coding sequence, whereas PrF and PrR indicate the positions of the forward and reverse primers, respectively. Following splicing, the corresponding complementary DNA fragments are 1,266 bp for *T. spiralis* and 870 bp for *T. nativa*. The expected amplified regions are 270 and 691 bp, respectively [99].

The most direct progression from molecular candidate selection to practical diagnostic assessment was achieved through comparative evaluation of recombinant serine proteases from *T. spiralis* (Figure 10A) and *T. nativa* (Figure 10B) [99].

In this comparative study, rTnsp-4E was identified as a promising diagnostic candidate for *T. nativa*, demonstrating substantial potential for early serological detection and emerging as a leading species-oriented antigen [25]. These advances complete a logical methodological progression, beginning with field confirmation of *T. nativa* circulation and extending to the preliminary evaluation of a species-specific diagnostic candidate.

Current evidence therefore supports a highly integrated methodological framework for diagnosing *T. nativa*. Long-term epizootiological monitoring confirms the natural circulation of the parasite and defines its geographic and host-associated risk profile. Hematological, biochemical, and serological investigations refine the early laboratory indicators of infection. Characterization of ES and somatic antigens, monoclonal antibodies, and stage-associated transcripts provides the basis for selecting diagnostically relevant molecules. Finally, bioinformatic evaluation and immunodiagnostic testing of alternative genes and recombinant proteins translate candidate discovery into species-oriented diagnostic strategies.

Collectively, these findings provide a strong methodological foundation for the subsequent development and field validation of assays designed not merely to detect trichinellosis, but to support the early and epidemiologically interpretable diagnosis of *T. nativa*.

## EVIDENCE GAPS AND FUTURE RESEARCH AGENDA

The principal evidence gaps concerning *T. nativa* arise not from the absence of diagnostic methods, but from the fact that the existing methodological framework remains largely derived from studies of *T. spiralis*. Consequently, it only partially reflects the distinctive biological characteristics of this cold-tolerant Arctic and subarctic taxon. Current limitations, therefore, extend beyond early detection and include the reproducibility of species identification, the interpretation of mixed infections, the comparability of geographically diverse datasets, and the validation of antigenic candidates developed specifically for *T. nativa* [19, 70, 97].

### Cross-reactivity and species specificity

A major unresolved issue is the extent of cross-reactivity between promising recombinant *T. nativa* antigens and other encapsulated taxa circulating in overlapping ecological systems and food chains, particularly *T. spiralis* and *T. britovi*. Despite the high diagnostic potential of candidates such as serine proteases, their practical value cannot be considered established without rigorous evaluation using sera from infections of confirmed species level etiology and samples representing naturally occurring mixed infections.

This requirement is reinforced by recent NGS findings demonstrating that co-infections involving multiple *Trichinella* taxa are not merely exceptional events but may represent a consistent component of Arctic and subarctic epizootiology [23, 98]. Diagnostic candidates must therefore be evaluated not only against unrelated helminths but also against closely related *Trichinella* taxa with overlapping antigenic profiles.

### Geographic and host-associated antigenic variation

Another important gap concerns the stability of the antigenic profile of *T. nativa* across geographically distinct populations and host species. For northern and Central Asian natural foci, comparable evidence remains insufficient to determine whether regional genetic variation alters antigen expression and, consequently, the sensitivity and specificity of serological candidates.

In this context, surveillance data from Kazakhstan are particularly relevant. Molecular confirmation of *T. nativa* circulation among wolves, foxes, and badgers establishes Central Asia as an important setting in which to evaluate the robustness of antigenic and molecular markers developed from a limited number of reference strains [10]. Future studies should therefore compare isolates from geographically separated Arctic, boreal, and Central Asian transmission systems and determine whether diagnostic performance is maintained across different wildlife hosts.

### Need for multicenter diagnostic validation

A further major limitation is the absence of comprehensive multicenter validation studies for *T. nativa*-specific diagnostic candidates. Transition from a promising experimental antigen to a standardized diagnostic tool requires a robust validation framework consistent with the recommendations of the International Commission on Trichinellosis and the World Organization for Animal Health.

Validation protocols for sylvatic parasites such as *T. nativa* must also account for the limited availability of well-characterized wildlife serum collections. Rather than depending exclusively on conventional large-cohort designs developed for widespread pathogens, prospective studies should be coordinated through national and international reference laboratory networks. Standardized repositories of confirmed positive and negative sera should be assembled to evaluate diagnostic sensitivity, specificity, repeatability, reproducibility, interlaboratory variation, and the distribution of optical density values used to establish assay cutoffs [12, 25].

Such studies should include sera from experimentally and naturally infected hosts, different stages of infection, a range of infection intensities, and infections caused by other *Trichinella* taxa and co-endemic helminths. Harmonized reporting of assay conditions and performance parameters would also improve comparability among studies.

### Balancing early detection and species specificity

A separate question is whether a diagnostically effective antigen panel for *T. nativa* can simultaneously shorten the serological window and improve species specificity. Available evidence indicates that a single molecular target is unlikely to satisfy both objectives consistently. Antigens expressed during the early enteral and migratory phases may improve early sensitivity but may not provide sufficient species selectivity. Conversely, proteins with stronger species specificity may be expressed later or elicit weaker antibody responses during the earliest stages of infection.

The most realistic strategy may therefore involve the development of multiplex antigen panels rather than pursuit of a single ideal antigen. Such panels could combine several recombinant proteins representing different developmental stages and antigenic functions. Their interpretation could then be supported by an algorithm incorporating the estimated stage of infection, clinical findings, exposure history, and regional epidemiological context [70, 89].

Future studies should evaluate whether combinations of serine proteases, ES-associated molecules, tyvelose-containing antigens, FATP1, PX-domain proteins, and other predicted targets improve diagnostic performance compared with individual antigens. Particular attention should be given to whether such panels can distinguish early infection from previous exposure and differentiate *T. nativa* from related encapsulated taxa.

### Optimization of wildlife meat surveillance

From an applied perspective, optimization of wild game meat surveillance remains unresolved, particularly within a One Health framework for regions where *T. nativa* represents the principal zoonotic risk. International standards emphasize that the sensitivity of artificial digestion depends strongly on tissue mass and selection of predilection muscles. Samples of up to 100 g may be examined when enhanced sensitivity is required.

In field settings, however, the optimal balance among laboratory capacity, sample volume, processing time, and the probability of missing low intensity infections has not been fully established for hunting systems in Arctic, boreal, and Central Asian regions. Species-specific differences in larval distribution among muscle groups further complicate the establishment of universal sampling protocols.

For *T. nativa*, detection of *Trichinella* spp. without subsequent species confirmation is also insufficient for interpreting risks associated with meat freezing, communicating with hunters and consumers, and determining the level of clinical vigilance required following exposure [10, 84]. Surveillance protocols should therefore integrate adequate tissue sampling, artificial digestion, estimation of LPG, and molecular confirmation of recovered larvae.

To support the rational allocation of resources according to immediate diagnostic, epidemiological, and biosecurity priorities, the remaining evidence gaps and corresponding research needs are systematically ranked in Table 2.

The future research agenda for *T. nativa* is closely linked to the standardization of high-resolution molecular identification. Although NGS provides substantial advantages for detecting mixed infections and identifying low-abundance or rare taxa, its widespread implementation requires standardized reference databases, harmonized species-classification criteria, contamination-control procedures, and reproducible bioinformatic workflows. Without such standardization, increasing analytical complexity may inadvertently introduce additional interlaboratory variability rather than improving diagnostic consistency [23, 97].

Accordingly, the next phase of diagnostic development should focus not simply on generating larger datasets but on establishing coordinated multicenter validation studies. Within these collaborative frameworks, species confirmation, quantitative assessment of diagnostic performance, standardized reporting of sensitivity and specificity, evaluation of mixed infections, and interlaboratory reproducibility should become mandatory components of study design [23, 97].

Overall, future research on *T. nativa* should progress from fragmented methodological advances toward an integrated, standardized diagnostic framework. Within this framework, early laboratory detection, species-specific serology, molecular confirmation, and epidemiological interpretation should function as complementary components of a unified diagnostic strategy rather than as independent approaches. Such integration is particularly important for Arctic, subarctic, and Central Asian ecosystems, where accurate species identification directly influences wildlife surveillance, food safety, outbreak investigation, and public health risk assessment, and where the consequences of diagnostic uncertainty remain substantial.

**Table 2:** Prioritized future research directions for T. nativa diagnostics and surveillance.

**Rank**	**Research priority**	**Primary objective**	**Required infrastructure and resources**	**One Health outcome**
1	Cross-reactivity validation of recombinant antigens (rTnsp-4E)	Evaluate species specificity against closely related *Trichinella* taxa, particularly *T. britovi* and *T. spiralis*	Well-characterized reference serum panels from experimentally and naturally infected hosts with confirmed single-species infections	Improved human and animal diagnostics through reduced false-positive results and more accurate differentiation of cold-adapted *Trichinella* species
2	Interlaboratory standardization of recombinant serological assays	Harmonize assay protocols, interpretation criteria, and diagnostic performance among regional and international reference laboratories	Standardized ELISA and Western blot reagents, calibrated instrumentation, reference sera, and external quality-assurance programs	Improved diagnostic harmonization through increased reproducibility, high interlaboratory agreement, and robust kappa-index performance
3	Optimization of artificial digestion protocols for wild game meat surveillance	Improve detection of low intensity infections caused by freeze-resistant *T. nativa* larvae in wildlife	Standardized World Organisation for Animal Health acid–pepsin digestion systems, optimized predilection-muscle sampling, and validated pooled-sample strategies	Enhanced food safety and veterinary surveillance through reduced foodborne transmission and optimized wildlife monitoring protocols
4	Development of comprehensive molecular reference libraries	Improve identification of mixed infections, rare genotypes, and emerging *Trichinella* variants	High-throughput sequencing platforms, curated reference sequence databases, standardized bioinformatic pipelines, and long-term sequence repositories	Improved environmental and wildlife surveillance through accurate mapping of sylvatic transmission, host-parasite dynamics, and geographic expansion

## CONCLUSION

This review provides a comprehensive synthesis of the current understanding of *Trichinella nativa*, integrating evidence from taxonomy, evolutionary biology, epidemiology, pathogenesis, diagnostics, and emerging molecular technologies within a One Health framework. The available literature consistently demonstrates that *T. nativa* is a biologically and epidemiologically distinct member of the genus *Trichinella*, characterized by exceptional freeze resistance, adaptation to Arctic–Subarctic ecosystems, and persistent circulation among wildlife reservoirs. Unlike other *Trichinella* species, its prolonged environmental survival and maintenance within sylvatic food webs present unique challenges for food safety, wildlife management, and public health, particularly in regions where the consumption of wild game meat is common.

The reviewed evidence indicates that advances in molecular epidemiology have substantially improved the understanding of *T. nativa* distribution and transmission dynamics. Long-term surveillance studies confirmed stable circulation in wild carnivore populations, while phylogenetic analyses, PCR-based assays, sequencing, and NGS have demonstrated that mixed infections involving *T. nativa* and other northern *Trichinella* taxa occur more frequently than previously recognized. These findings challenge the traditional assumption of single-species infections and highlight the complexity of parasite transmission within Arctic and boreal ecosystems. Similarly, advances in immunological research have identified promising recombinant antigens, particularly serine protease-derived candidates such as rTnsp-4E, which show encouraging potential for shortening the serological window and improving the early detection of infection.

From a practical perspective, the evidence supports a transition from conventional single-method diagnosis toward integrated diagnostic algorithms. Artificial digestion remains indispensable for larval recovery and food safety inspection, whereas multiplex PCR, confirmatory sequencing, and NGS collectively provide increasingly accurate species identification, detection of mixed infections, and molecular epidemiological surveillance. Such integrated diagnostic strategies are particularly important for *T. nativa*, because species level identification directly influences risk assessment for frozen game meat, outbreak investigations, wildlife surveillance, and evidence-based public health recommendations. The review also reinforces the importance of coordinated One Health surveillance that links veterinary inspection, wildlife monitoring, environmental surveillance, and human health systems to reduce zoonotic transmission risks.

A major strength of the current body of evidence is the progressive integration of classical parasitology with modern molecular biology, genomics, immunology, and bioinformatics. Long-term wildlife surveillance, comparative genomic analyses, recombinant antigen development, and high-resolution sequencing technologies have collectively transformed the understanding of *T. nativa* ecology and diagnostics. These multidisciplinary advances provide a strong scientific foundation for developing more sensitive, species-specific, and epidemiologically informative diagnostic tools.

Nevertheless, important limitations remain. Much of the available diagnostic knowledge continues to rely on extrapolation from *T. spiralis*, while validated *T. nativa*-specific diagnostic reagents, multicenter validation studies, and geographically representative antigenic datasets remain limited. Information on antigenic variability among regional populations, cross-reactivity with closely related *Trichinella* species, standardized NGS analytical pipelines, and reproducible reference materials remains insufficient. Furthermore, the limited availability of well-characterized wildlife serum collections and naturally infected field samples continues to constrain large-scale validation of promising diagnostic candidates.

Future research should therefore prioritize the development and international validation of *T. nativa*-specific recombinant antigens, standardized multiplex serological platforms, harmonized molecular identification protocols, and comprehensive genomic reference libraries. Large multicenter studies involving wildlife, domestic animals, and human samples are required to establish robust diagnostic performance, evaluate mixed infections, and determine the influence of geographic genetic variation on antigenicity. Simultaneously, advances in portable molecular technologies, high-throughput sequencing, artificial intelligence-assisted bioinformatics, and integrated One Health surveillance networks offer promising opportunities to improve early detection, outbreak investigation, and real-time monitoring of this Arctic parasite.

In conclusion, *T. nativa* should no longer be regarded simply as another member of the genus *Trichinella*, but as a distinct zoonotic pathogen requiring dedicated diagnostic, epidemiological, and surveillance strategies. Continued integration of conventional parasitological methods with recombinant immunodiagnostics, high-resolution molecular technologies, standardized international validation frameworks, and coordinated One Health surveillance will be essential for improving food safety, strengthening wildlife disease monitoring, and reducing the public health burden associated with this uniquely cold-adapted parasite. Such an evidence-based and internationally harmonized approach represents the most effective pathway toward accurate diagnosis, improved risk assessment, and sustainable control of *T. nativa* in Arctic, Subarctic, and Central Asian ecosystems.

## DATA AVAILABILITY

All the generated data are included in the manuscript.

## GENERATIVE AI DECLARATION

Google Gemini was used solely to create the figures. AI was not used for text preparation, data analysis, or interpretation of the results.

## AUTHORS’ CONTRIBUTIONS

OA: Conceptualization, study design, project administration, supervision, and writing–original draft preparation. AZh: Review design, data analysis, and manuscript review. AG: Writing–original draft preparation and formal analysis. SI: Data analysis and writing–original draft preparation. AS, NG, and AI: Data collection, data curation, and data analysis. FZh: Data interpretation, manuscript review, and editing. NA and AJ: Literature search, data analysis, and evidence synthesis. CPR: Methodological guidance, data extraction, data analysis, and critical manuscript review. ZhA: Conceptualization and review design. AM: Literature search and evidence collection. All authors have read and approved the final version of the manuscript.
